# Metabolic profiling of human brain metastases using in vivo proton MR spectroscopy at 3T

**DOI:** 10.1186/1471-2407-7-141

**Published:** 2007-07-27

**Authors:** Torill E Sjøbakk, Roar Johansen, Tone F Bathen, Ursula Sonnewald, Kjell A Kvistad, Steinar Lundgren, Ingrid S Gribbestad

**Affiliations:** 1 Department of Neuroscience, Norwegian University of Science and Technology (NTNU), Norway; 2 Department of Cancer Research and Molecular Medicine, NTNU, Trondheim, Norway; 3 Department of Circulation and Medical Imaging, NTNU, Trondheim, Norway; 4 Department of Radiology, St. Olavs University Hospital, Trondheim, Norway; 5 Department of Oncology, St. Olavs University Hospital, Trondheim, Norway

## Abstract

**Background:**

Metastases to the central nervous system from different primary cancers are an oncologic challenge as the overall prognosis for these patients is generally poor. The incidence of brain metastases varies with type of primary cancer and is probably increasing due to improved therapies of extracranial metastases prolonging patient's overall survival and thereby time for brain metastases to develop. In addition, the greater access to improved neuroimaging techniques can provide earlier diagnosis. The aim of this study was to investigate the feasibility of using proton magnetic resonance spectroscopy (MRS) and multivariate analyses to characterize brain metastases originating from different primary cancers, to assess changes in spectra during radiation treatment and to correlate the spectra to clinical outcome after treatment.

**Methods:**

Patients (n = 26) with brain metastases were examined using single voxel MRS at a 3T clinical MR system. Five patients were excluded due to poor spectral quality. The spectra were obtained before start (n = 21 patients), immediately after (n = 6 patients) and two months after end of treatment (n = 4 patients). Principal component analysis (PCA) and partial least square regression analysis (PLS) were applied in order to identify clustering of spectra due to origin of metastases and to relate clinical outcome (survival) of the patients to spectral data from the first MR examination.

**Results:**

The PCA results indicated that brain metastases from primary lung and breast cancer were separated into two clusters, while the metastases from malignant melanomas showed no uniformity. The PLS analysis showed a significant correlation between MR spectral data and survival five months after MRS before start of treatment.

**Conclusion:**

MRS determined metabolic profiles analysed by PCA and PLS might give valuable clinical information when planning and evaluating the treatment of brain metastases, and also when deciding to terminate further therapies.

## Background

Brain metastases are a common oncologic challenge as approximately 15–20 % of all cancer patients develop central nervous system metastases. They are the dominating type of brain tumor and are most frequently caused by haematogenous spread from a primary cancer in lung, breast, skin or colon [1]. Survival varies with type and grade of primary cancer, age at diagnosis as well as extent of proliferation of the primary cancer [2]. The overall prognosis of survival for these patients is generally poor and the management of the disease is a significant challenge.

A major goal in oncology treatment is to give each patient an individualized therapeutic regime. In addition to diagnosis, the patient's pre-treatment performance status described by Karnofsky performance score (KPS) and the recursive partitioning analysis (RPA) is evaluated [3]. The standard treatment is whole-brain-radiation-therapy (WBRT) alone or after surgery which may prolong life from one to nine months [4]. Radiation therapy is the most common treatment strategy since more than 70 % of the patients have multiple metastases at the time of diagnosis [5]. Chemotherapy may also be used in selected patients. To decide between different treatment options various prognostic factors such as age, performance status, numbers of brain metastases and type of primary cancer and extent of extracranial metastases activity are evaluated. Some patients get their brain metastases detected before the primary cancer and a non-invasive identification of type of metastases would be of importance for further treatment [6]. In order to optimize and avoid ineffective treatment there are also a need for early evaluation of response. The diagnosis and planning of brain tumor treatment have become more advanced due to improved diagnostic neuroimaging tools. One of these is in vivo magnetic resonance spectroscopy (MRS) which provides additional information for classifying most brain tumor types and grades [7-9].

Proton (^1^H) in vivo MRS can be used to quantify metabolites and monitor response to therapy in brain tumors, thereby allowing non-invasive monitoring of tumor biochemistry [7]. Previous studies have used 1.5T systems for classification and characterisations of brain tumors. Recent clinical instrument operating at 3T has improved spectral quality related to resolution and signal-to-noise-ratio [10-12]. The main metabolites in proton spectra of normal brain tissue are N-acetyl aspartate (NAA), creatine (Cr) and choline-containing compounds (tCho) at 2.0, 3.0 and 3.2 ppm, respectively. In brain malignancies lipid signals at 1.3 (methylene) and 0.9 ppm (methyl) appear when short echo times are used [8,13]. Previous studies have addressed metabolic changes in primary brain tumors occurring after radiation therapy [14-16]. The reports from different groups are not conclusive but reduction in tCho appears to be a reliable marker for treatment response. Among the parameters being predictive for poorer outcome in certain patient sub-groups were higher tCho/Cr and tCho/NAA ratios and higher lipid signals and lower Cr/NAA ratio [17]. Less is known about brain metastases and the objectives of this study were to characterize brain metastases originating from different primary cancers, to assess changes in spectra during radiation treatment and to correlate the spectra to clinical outcome for the patients after treatment. Our hypothesis is that in vivo MRS from different brain metastases analysed by multivariate analyses distinguish metastases originating from different primary tumors, and also that MR spectra can indicate clinical outcome for these patients.

## Methods

### Patients

A total of 26 consecutive patients (18 women and 8 men) with brain metastases from primary cancers such as breast (n = 9), lung (n = 9), malignant melanoma (n = 4), colon (n = 3) and kidney (n = 1) were enrolled in this study. All patients gave a written informed consent and the study was approved by the local ethics board; Central Norway medical reach ethics committee. After contrast enhanced MRI, metastases larger than 10 mm in diameter were chosen for MRS. The quality evaluation of each MR spectrum, led to the exclusion of five of the 26 enrolled patients (one from each primary cancer group). A description of the 21 remaining patients is given in Table 1. Six patients came to a second examination immediately after completed radiation treatment and four patients to a third MRI/MRS examination after additional two months. The same size of volume of interest (VOI) was used in each of the repeated examinations. The patients' performance status (KPS and RPA) was determined before start of treatment.

**Table 1:** Patient characteristic. Treatment and clinical outcome data for all patients with approved MR spectra.

**Patient no.**	**Sex, Age**	**Primary cancer**	**Brain mets**	**Mets other sites than brain**	**KPS**	**RPA**	**Survival (months)**	**Treatment**	**Time: diagnosis- 1.MRS (weeks)**	**Spectral data TE, VOI**^1,2^
**1**	F, 52	Breast	Single	Brain only	90	1	13+	S + 3Gyx10	5	32,144^1^
**2**	M, 44	Lung	Single	Brain only*	90	1	10+	S	1	33,144^2^
**3**^a^	F, 47	Lung	Multiple	Brain only	90	1	7+	C	0	32,144^1^
**4**^a^	F, 45	Breast	Multiple	Mediastinum	100	2	15+	S + 3Gyx10	1	33,144^2^
**5**^a^	F, 48	Breast	Multiple	Skeleton*	100	2	5+	3Gyx10	0	33^2^
**6**	F, 57	Lung	Multiple	Intestinal, jejunum	100	2	7	3Gyx10	4	32,144^1^
**7**	M, 63	Lung	Multiple	Lung, skeleton	100	2	6	3Gyx10	2	32,144^1^
**8**	M, 62	Lung	Single	Skeleton	90	2	4.5	S + 3Gyx10	4	33,144^2^
**9**	M, 70	Mal. mel.	Multiple	Lung, subcutan*	90	2	2	S + 3Gyx10	3	33 in 2 VOI^2^
**10**	F, 36	Breast	Multiple	Liver, skeleton	80	2	9	4Gyx5 (GK)	1	32,144^1^
**11**	F, 47	Breast	Multiple	Liver	80	2	10	3Gyx10	2	32,144^1^
**12**^b^	F, 66	Breast	Multiple	Brain only	80	2	2	3Gyx10	3	33 in 2 VOI^2^
**13**	M, 70	Colon	Single	Liver, lung	80	2	6	S + 4Gyx5	17	33,144^1^
**14**^b^	F, 80	Lung	Multiple	Brain only	80	2	0.75	4Gyx5	1	33 in 2 VOI^2^
**15**^a^	F, 71	Lung	Multiple	Brain only	80	2	3	3Gyx10	4	33 in 2 VOI^2^
**16**	F, 72	Breast	Multiple	Liver, skeleton	60	3	0.5	4Gyx5	2	33,144^2^
**17**	M, 69	Colon	Single	Liver, lung	60	3	6	S + 3Gyx10	5	32,144^1^
**18**^a^	F, 53	Lung	Multiple	Brain only*	60	3	11+	4Gyx5	2	33 in 2 VOI^2^
**19**	M, 56	Mal. mel.	Multiple	Epigastrium	60	3	1	No Treatment	2	33^2 ^and 32^1^
**20**	F, 63	Breast	Single	Lung, skeleton, lymph node	50	3	0.5	No treatment	0	33,144^2^
**21**	F, 71	Mal. mel.	Single	Lung, liver	50	3	1.5	3Gyx10	8	32,144^1^

F = female, M = male, mets = metastases, ^a^: three MRS follow-up examinations, ^b^: two follow-up MRS examinations, *: brain metastases detected before primary cancer, KPS = Karnofsky Performance Score, RPA = Recursive Partitioning Analysis classification, TE = echo time, VOI^1,2 ^= volume of interest, ^1^: voxel size: 15 × 15 × 15 mm^3^, ^2^: 10 × 10 × 10 mm^3^. Mal. mel. = malignant melanoma, GK = gamma knife, S = Brain surgery and histopathologic verification, C = Chemotherapy.

### In vivo MR spectroscopy

MR imaging and spectroscopy examinations were performed using a 3T clinical MR system (Philips Intera, Best, The Netherlands) equipped with a transmit-receive head coil. The MRI protocol consisted of conventional T_1 _– and T_2 _– weighted images before and after an intravenous injection of 0.2 ml Gadodiamide/kg body weight (Omniscan™, GE Healthcare). Single voxel ^1^H MRS was performed after contrast injection on a VOI localized within the metastases using the point resolved spectroscopy (PRESS) pulse sequence, repetition time (TR) of 2000 ms and echo time (TE) of 32/33 ms. When only one metastasis (> 10 mm) was detected, a second MRS acquisition was performed of the same volume using TE = 144 ms (18 patients). If two or more large metastases were identified by the MRI, a second spectrum was obtained using the short TE in one of the other tumors (7 patients). The VOI was 15 × 15 × 15 or 10 × 10 × 10 mm^3^, depending on the size of the actual metastasis, and all localizations were verified by an experienced neuroradiologist. Bandwidth, number of points and sampling interval were 2000 Hz/1024/0.5 ms. Each spectrum was obtained as an average of 192 measurements, giving an acquisition time of 7 minutes. Shimming (automatic) prolonged the acquisition time with about three minutes. A routine water unsuppressed spectrum (16 measurements) obtained at each examination was used to evaluate the spectrum quality. A total of 33 spectra with short and 18 spectra with long TE were obtained from brain metastases during the MRS examinations. The spectroscopic data were post-processed using jMRUI [18]. The FIDs were zero-filled (2 K) and a Lorentzian filter (2 Hz) was applied before Fast Fourier Transformation (FFT). The residual peak of the water signal was suppressed using Hankel Lanczos Singular Values Decomposition Filter (HLSVD)[18].

### Data analyses

The spectral quality was evaluated by applying the algorithm AMARES to estimate the linewidth (full-width-half-maximum, FWHM) of the water peak signal in the corresponding water unsuppressed spectra. A mean FWHM ± SD of water peaks in all spectra was calculated (9.2 ± 2.1 Hz). The mean value with the corresponding positive 99% CI (2.1 Hz) was chosen as quality criteria. All spectra with a higher FWHM than 11.3 Hz were excluded from further data analyses. Spectral data were transferred to the software program Unscrambler (CAMO) as ASCII-files, Baseline offset was adjusted and the lipid signal at 1.3 ppm was used as a chemical shift reference. The spectra were mean normalised and examined using principal component analysis (PCA) and partial least square regression analysis (PLS). Only the chemical shift region including the resonances from lipids to tCho compounds (0.70 – 3.45 ppm/365 spectral points) was investigated. Mean spectra for each primary cancer group were made to illustrate the spectral variations.

PCA was applied in order to identify clustering of spectra due to origin of metastases based on examination of score plots and loading profiles. The PCA model compresses or simplifies high-dimensional data by finding a linear combination of the original variables so the variance is maximized and new uncorrelated variables, principal components (PC) are created. To avoid that the model described too much of the variation in the data i.e. noise, the number of PCs to retain in the model was kept as low as possible. PCA was performed with full cross validation and mean centering.

The PLS was applied in order to relate clinical outcome of the patients (survival or not at five months after first MR examination) to the spectral data (obtained at first MR examination). PLS is the regression extension of the PCA and the data are reduced into PLS factors which explain most of the variation in both predictors (MR spectra) and responses (clinical parameters). The number of PCs to remain in the model was determined by finding the PC where the total residual y-variance and root mean square error of prediction (RMSEP) were minimized. For the PLS analysis the test set method was used as validation method. The test samples were selected in advance by using the Kennard Stones algorithm for splitting data sets into two subsets [19], resulting in 14 samples for calibration and 7 samples for testing. The significance of the estimated correlation factors between measured and predicted y-variables was ascertained by using the Pearson correlation test (two-tailed).

## Results

Of the 33 short echo time spectra obtained from the enrolled 26 patients, six spectra were excluded due to a FWHM of the water signal larger than 11.3 Hz, likewise four of the eighteen spectra obtained at TE 144, leaving 27 spectra with short and 14 with long echo time for further analyses.

**Figure 1:**
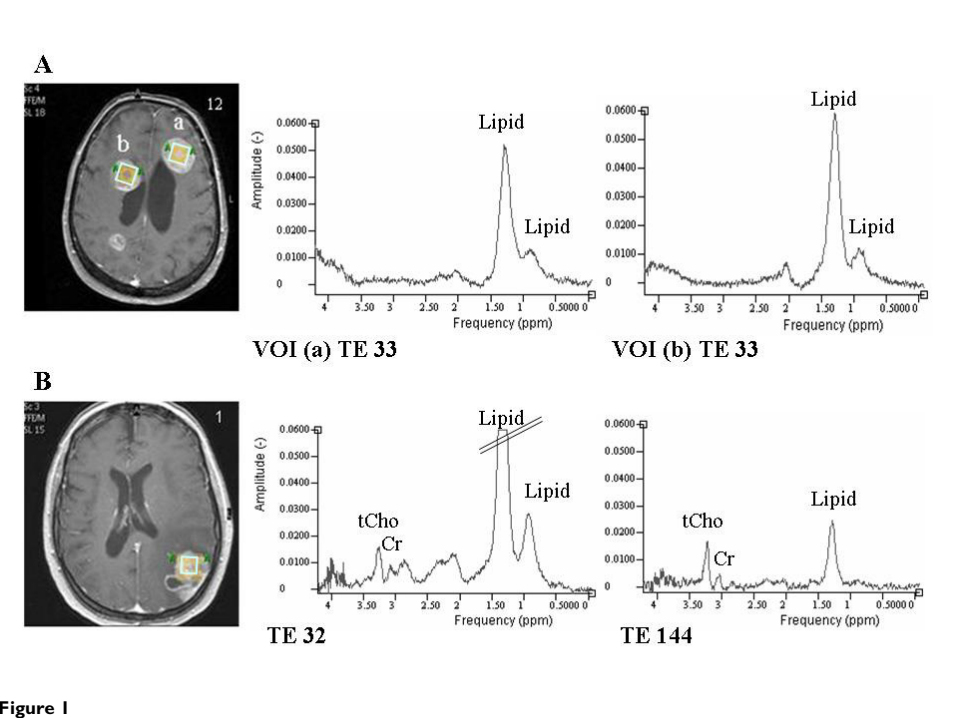
**Brain metastases in vivo spectra**. Axial T1 weighted contrast enhanced MR images of two patients with brain metastases from breast cancer (patient 1 and 12) with corresponding spectra. A: short echo time spectra of two metastases (a and b). B: Short and long echo time spectra in the same VOI. The lipid peak at 1.3 ppm in the short echo time spectrum is cut due to the chosen scaling.

**Figure 2:**
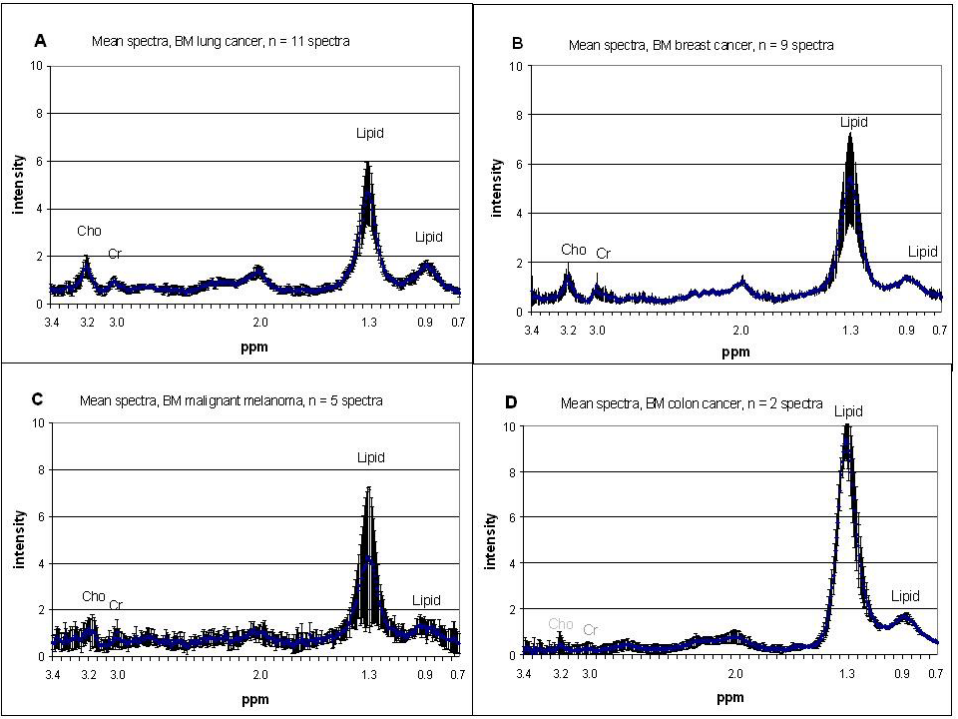
**Mean spectra**. Short echo time mean spectra ± 95% CI of brain metastases from different primary cancer. A: lung cancer (n = 11 spectra), B: breast cancer (n = 9 spectra), C: malignant melanoma (n = 5 spectra), D: colon cancer (n = 2 spectra). The 0.7 – 3.4 ppm area of the spectra and the detected metabolites (in ppm) are given; tCho (3.2), Cr (3.0): creatine and lipids (1.3, 0.9): methylene and methyl groups.

Spectra from two distinct metastases were obtained in six patients (Table 1). Typical axial T1 weighted contrast enhanced MR images with corresponding in vivo spectra of metastases in two breast cancer patients are given in Fig. 1. Mean spectra (short TE) ± 95% CI of the four different primary cancer groups are shown in Fig. 2. The lipid signals at 1.3 and 0.9 ppm were the dominating peaks in the majority of the spectra in all groups. Cr, tCho and a broad peak around 2.0 – 2.2 ppm were also detected in 17, 20 and 22 of the 27 spectra, respectively.

**Figure 3:**
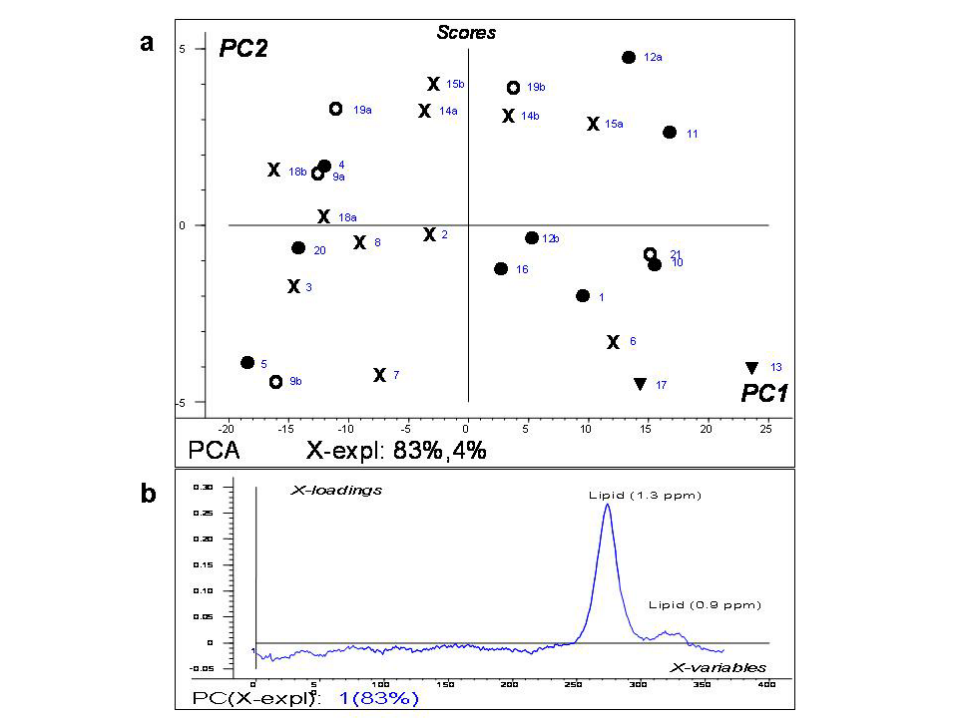
**PCA results**. Part a: Score plot of PC1 versus PC2 of water suppressed in vivo spectra. ● = breast cancer (n = 9), **X **= lung cancer (n = 11), **O **= malignant melanoma (n = 5), ▼ = colon cancer (n = 2). Part b: The loading profile of PC1 showing differences in the lipid signals.

The score plot from PCA of the 27 short echo time spectra obtained before start of treatment is presented in Fig. 3a. This plot indicates that brain metastases from primary lung and breast cancer tend to cluster, while the metastases from malignant melanomas show no uniformity. The two spectra of metastases from colon cancer are found in the lower right quadrant of the score plot. The separation of breast and lung metastases is based on PC1 and PC2, which account for more than 83 % of the total variation of the spectra. Samples with high score for PC1 are characterized by high lipid signal and no other metabolites, as described by the loading profile of PC1 (Fig. 3b).

**Figure 4:**
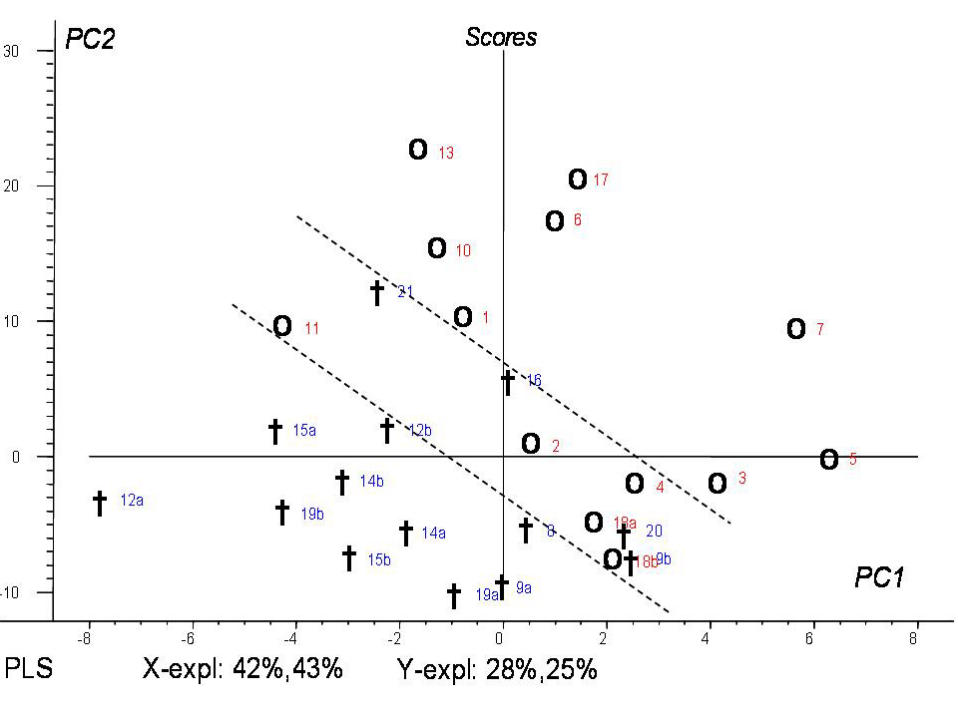
**PLS score plot**. Score plot of spectra from patients who lived longer than five months (**○**), and they who passed away before five months after the first MR spectra examination (**†**). The numbers refers to patient numbers in Table 1. Some patients were examined for two metastases (a and b).

Figure 4 gives a score plot of PC1 versus PC2 from the PLS relating spectra of untreated metastases to survival five months after treatment. The first two PCs explained totally 85 % of the x-variables (the spectral data) and 53 % of the y-variable (survival). The correlation factor between measured and predicted survival were 0.73 (p < 0.01) for the calibration run and 0.76 (p < 0.05) for the corresponding validation (seven test samples). The spectra of patients who survived five months after first MRS (marked as circles) are clustered on the right side of the score plot, while the spectra of patients who survived less than five months (cross marks) are clustered in the opposite side.

**Figure 5:**
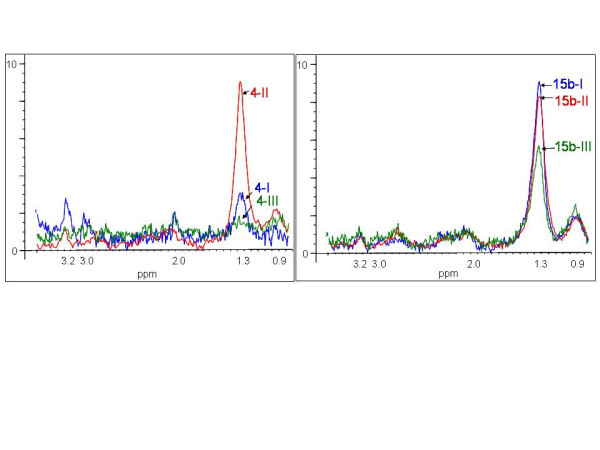
**Follow-up spectra**. Spectra of brain metastases from patients with primary breast (patient 4) and lung cancer (patient 15), before (-I), immediately after (-II) and two months after end of radiation treatment (-III). The patients' survival was more than 16 months and only 3 months, respectively.

Follow-up MRS examinations where completed only for four of the included patients, since some got a complete surgical resection of the metastasis, a few refused further examinations and some died shortly after the first or second examination (Table 1). Two examples of the follow-up spectra are shown in Fig. 5. At the last examination four of the seven obtained spectra (four patients) showed decreased levels of the lipid signal (30–80 % peak intensity of mean normalized spectra). Of the ten second examination spectra six showed minor changes in the metabolite distribution, while three spectra indicated an increase in the 1.3 ppm lipid peak relative to the signal of methyl at 0.9 ppm. The tenth spectra, obtained from the patient treated with chemotherapy showed an increasing signal of NAA and Cr peak relative to Cho-containing signals at 3.2 ppm indicating normal brain tissue enclosed in the VOI due to the shrinking metastasis during the treatment period.

## Discussion

The results presented here demonstrate that the lipid signals are important in brain metastases characterization. The clustering of spectra from brain metastases from breast and lung cancer in the PCA score plot is described by PC1 showing the lipid signal as the dominating difference. The lipid signals are also an important factor in the PLS relating MR spectra to clinical outcome. These signals change after radiation therapy, which is in accordance to previous studies [14,16]. Many cellular processes such as proliferation, inflammation, malignancy, necrosis, growth arrest and apoptosis, have been linked to alterations in MR visible lipid levels. Intracellular lipid bodies appear to be the most important contributors [20,21]. However, which biochemical processes that lead to lipid accumulation of MR visible signals is still enigmatic, and the source and complete characteristics of the lipids found mostly in high grade brain tumors are still not elucidated [21,22].

Small signals of intracellular lipids might be observed in normal brain tissue in short echo time spectra and these signals are often increased in malignancies. In accordance with previous studies of brain tumors [8,14], increased signals of lipids and also tCho compounds were detected in the majority of all spectra of brain metastases obtained with short echo time in this study (Fig. 2). The other main metabolites in spectra from normal brain tissue, NAA and Cr were definitely reduced in these spectra. The high intensity lipid signals and low levels of Cr are typical for metastases and have been used to differentiate these lesions from primary brain tumors [22-25]. However, the spectral differences between metastases and high grade gliomas are small and these two groups might be hard to distinguish [23]. Methylene components in fatty acids give rise to a broad peak around 2.0 – 2.2 ppm [21] and this might overlap any possible NAA present. At longer echo time the lipid signals will be reduced due to short relaxation time [26], and in seven of the fourteen spectra obtained at TE 144, a small peak of NAA could be observed. Previous studies have interpreted that signal from NAA in metastases is due to the presence of viable neurons within an infiltrative tumour or contamination from normal brain tissue since NAA is a neuron specific metabolite [24,27].

The use of multivariate analyses can reveal features in the spectra which may not be detectable with traditional statistical analyses of peak integral ratios [28,29]. The mean spectra from the different primary cancer groups showed minor differences in distribution of the metabolites. Still, the spectra from metastases in breast and lung cancer patients tend to cluster into different parts of the PCA score plot, the separation being mainly due to differences in the lipid signals as described by PC1 (Fig. 3b). The dispersion of the malignant melanoma spectra in the PCA score plot (Fig. 3a) reflects the large confidence interval for the corresponding averaged spectra (Fig. 2c).

In this study, PLS was used to relate spectral data to clinical outcome for each patient. A trend of two separate clusters of patients surviving five months versus patients passing away before five months can be observed in the score plot (Fig. 4) The loading weights from PLS indicate that resonances in the ppm regions 0.8 – 0.9, 1.8 – 2.2 and 2.5 – 2.8 ppm representing different lipid signals are most important for the prediction. By seeing the patients' performance status in conjunction with the respective localization in the score plot (Fig. 4), patients classified as RPA2 and 3 are dispersed across the plot, while the RPA1 classified patients are clustered in the intermediate area. Patients classified as RPA3 might be interpreted to have a better prognosis if the spectral data appear to be in the survival area of the score plot. Thus, the use of multivariate analysis on the spectral data might be of importance to predict the outcome for patients with brain metastases.

Half of the patients in this study received radiation therapy and the follow-up spectroscopy examinations were done immediately after completed treatment. However, only a few of these patients were able to fulfil the study protocol due to their health condition. The changes in level of mobile lipids and choline observed in spectra obtained after completed radiation therapy could reflect effects of the treatment observed at a metabolic level. Graves et al. [30] have previously shown changes in the metabolic profile of recurrent gliomas up to 14 months after gamma knife surgery. They observed an increase in the lactate/lipid resonance that developed early after the treatment but was reduced later in the follow-up of these glioma patients. Nelson [31] reported that the intensity of the tCho, Cr, and NAA signal decreased with time after radiation treatment of gliomas, and the lactate/lipid ratio increased to a maximum four months after the end of radiation. This is interpreted to represent a reduction in tumor and formation of treatment-induced necrosis. Possibly, the changes observed in the present study of brain metastases could be caused by the same mechanisms.

Single voxel spectroscopy has been extensively used in examination of brain tumors [8,22,24,26] and has been included in routine MRI protocols for tumor diagnostics at several sites. Differentiation of most tumor types has been successfully reported and a clinical decision support system for classification of brain tumors, INTERPRET, has recently been developed [32]. The short acquisition time makes it simple to include it in a clinical MRI exam and the 3T generation of MR instruments will give better spectral resolution and signal-to-noise ratio with equal effective size of VOI [12]. The spatial resolution will, however, be poor compared to MR Spectroscopic Imaging (MRSI). Using MRSI, several single voxels over a wider area become examined at the same time and metabolite distribution in both tumor and surrounding normal tissue are explored. However, single voxel MRS is less time consuming both during optimization and acquisition than MRSI. The signal from the whole metastasis was used for studying the metabolic phenotype in these analyses.

The exclusion criterion used in this study was the mean FWHM value for the corresponding unsuppressed water signal of the obtained spectra. This threshold value should not be considered as an exact threshold normative for other studies due to the small group investigated. However, the method of excluding spectra that are outside the 99% confidence interval of the data could be a used as a standardized quality control. The exclusion criterion excluded five of the enrolled twenty-six patients. Due to their bad health condition, some of the patients where uncomfortable during the examination which could cause motion artefacts, while in three of the patients the metastases were localized near the skull or close to cerebellum. The use of contrast agent has shown no significant effect on metabolite signal intensities at 1.5T [33,34]. The effect of the contrast agent has not been tested in this study. However, for clinical MRS studies of brain metastases in vivo the contrast agent will always be present as a part of the routine MRI protocol.

Spectrum quality also depends on the voxel size. The spectral SNR increases linearly with the voxel size and with the square root of the number of acquisitions [35]. To obtain maximum SNR within acceptable examination time, the number of acquisitions used was chosen as many as possible, resulting in 192 acquisitions during a seven minutes examination. In about half of the patient group, the smallest possible voxel size was used due to the size of the metastasis. Hence, these cases should give reduced SNR compared to the other cases where the cubic sides where 15 mm. Among the excluded spectra two out of five where obtained using the largest volume size.

A clear limitation of the present study is the small and heterogeneous group of patients and increased number of patients to further validate the clinical value of the PLS model is necessary. A possible bias could be the skewed distribution of the subgroups of brain metastases. There were twice as many patients with brain metastases from breast and lung cancer as the other groups. This reflects however the incidence of brain metastases from the different type of primary cancer [1].

## Conclusion

The in vivo metabolic MR profiles of brain metastases demonstrated variations due to origin of primary cancer and as an effect of radiation treatment. A correlation between MR profiles and survival at five months was also found. Thus, MR determined metabolic profiles might contain valuable clinical information for planning and evaluation of brain metastasis treatment.

## Competing interests

The author(s) declare that they have no competing interests.

## Authors' contributions

The study was conceived by ISG, SL and US while all authors contributed to the design of it. SL and RJ included the patients and provided the clinical information. TES organized the MRS examinations, performed the data analyses and wrote the paper. KAK were responsible for the MRI and MRS examinations. TFB contributed with multivariate analyses and discussions. All authors discussed, read and approved the final manuscript.

## Pre-publication history

The pre-publication history for this paper can be accessed here:


